# Prevalence and Risk Factors Associated with Postpartum Depression during the COVID-19 Pandemic: A Literature Review and Meta-Analysis

**DOI:** 10.3390/ijerph19042219

**Published:** 2022-02-16

**Authors:** Qianqian Chen, Wenjie Li, Juan Xiong, Xujuan Zheng

**Affiliations:** Health Science Centre, Shenzhen University, Shenzhen 518060, China; 1800243042@email.szu.edu.cn (Q.C.); 2019227013@email.szu.edu.cn (W.L.)

**Keywords:** review, postpartum depression, COVID-19, prevalence, risk factors

## Abstract

Background: Owing to the high prevalence and detrimental consequences, postpartum depression (PPD) has been identified as one of the severe global public health issues in the last decade. Prior research found that during disasters or events, the prevalence rates of mental disorders among postpartum women are significantly high. However, the effect of the coronavirus disease 2019 (COVID-19) pandemic on PPD and its risk factors remained unclear for postpartum women. Therefore, the present systematic review and meta-analysis aimed to estimate the influence of the COVID-19 pandemic on the prevalence of PPD and to summarize risk factors for PPD during the COVID-19 pandemic. Methods: Three electronic databases of MEDLINE, EMBASE, and Cochrane library databases were systematically searched for articles from their commencements until 1 November 2021. Quality assessment of included studies, random-effects meta-analysis, and sensitivity analysis were performed. Results: A total of eight studies with 6480 postpartum women during the COVID-19 pandemic were included, and most studies were conducted in developed countries. The pooled prevalence of PPD was 34% (95% CI: 21–46%) during the COVID-19 pandemic, much higher than the incident of previous research during the non-pandemic period. Risk factors for PPD during the COVID-19 pandemic were defined as socio-demographic and clinical characteristics, stress and anxiety, lack of various supports, and the COVID-19 related factors. Conclusion: The research findings indicated that the COVID-19 pandemic could make detrimental effects on maternal mental wellbeing among women after childbirth. Investigating the prevalence and risk factors of PPD among postpartum women could shed some light on their mental and emotional states; so that support measures and tailored interventions from health professionals and policymakers could be offered to improve the maternal and infant outcomes, especially during the COVID-19 pandemic. Much more research on maternal psychological wellbeing during the COVID-19 pandemic was strongly recommended to undertake in the middle and low-income countries.

## 1. Introduction

The transition of motherhood is a challenging period during which women need to acquire various parenting knowledge and skills, adapt to new family relationships, and experience immense physical and psychological changes [1], making them vulnerable to mental disorders [2]. Postpartum depression (PPD) or postnatal depression (PND), as one of the most common mood disorders of women after childbirth [3], refers to any major or sub-clinical depression that women have within the first year postpartum [4]. Women with PPD can suffer from some common symptoms, such as sleep disorder, irritability, poor appetite, loss of interests, low self-respect, and some women with PPD even have thoughts of harming their child and self-harm [5,6,7].

Previous studies have reported that the worldwide incidence of PPD varied from approximately 9.5% in high-income countries, about 20.8% in middle-income countries, and to 25.8% in low-income countries until 2017 [8,9]. It needs to be noted that most women with PPD may recover within a few months, but about 30% of women could have depression beyond the first year after childbirth [10]. Furthermore, the risk of recurrence was approximately 40% in either subsequent postpartum or non-postpartum. In addition to the increasing incidence, PPD was identified to negatively influence the physical and mental wellbeing of women [7], the behavioral and emotional development of infants [11], and the relationship with spouse and other family members [12]. Owing to the high prevalence and detrimental consequences, PPD has been identified as one of the severe global public health issues in the last decade [7,13].

In January 2020, the World Health Organization (WHO) declared the novel coronavirus disease 2019 (COVID-19) as an international public health emergency; and in March 2020, it was raised to a global pandemic [14,15]. In order to limit the spread of COVID-19, Public Health and Social Measures (PHSM) strongly recommended by WHO were conducted in all countries of the world, such as restrictions on public and private gatherings [16]. Previous studies on similar epidemics, i.e., SARS (Severe Acute Respiratory Syndromes) and MERS (Middle East Respiratory Syndrome Coronavirus) have reported that social isolation and health service disruptions could detrimentally affect the mental health and psychological wellbeing of people [17,18,19]. Prior research likewise found that during disasters or events, the prevalence rates of mental disorders among perinatal women are significantly higher than those among the general population [20]. Regarding that the previous review was based on studies from previous epidemics, the research findings were not specific to COVID-19 [21]. Amid the COVID-19 pandemic, the psychological wellbeing of women in the postpartum period would be at stake but often overlooked [21]. Therefore, the effects of COVID-19 on the prevalence of PPD and its risk factors needed to be explored, and applying tailored interventions on the basis of data is in urgent need [20]. 

There were three meta-analytic reviews reporting maternal mental health during the COVID-19 pandemic [20,21,22]. However, most of the study samples in the three reviews were pregnant women; all articles were searched until 2020 using highly diversified tools of PPD and cut-off scores, and the risk factors of PPD have not been involved. Thus, the present systematic review and meta-analysis aimed to estimate the influence of the COVID-19 pandemic on the prevalence of PPD and to summarize and determine risk factors for PPD during the COVID-19 pandemic.

## 2. Materials and Methods

### 2.1. Search Strategy

The literature was searched with MEDLINE, EMBASE, and Cochrane library databases. We searched with the following terms: (postpartum OR post-birth OR post birth OR perinatal OR postnatal OR puerperium) AND (prevalence OR incidence) AND (postpartum depression OR depression OR depressive OR depressed OR depress * OR mental health) AND (factor * OR risk OR indicator OR refer * OR indication OR screen * OR predict *) AND (pandemic OR crisis OR epidemic OR outbreak OR COVID OR coronavirus OR lockdown OR quarantine). All articles were published in English from inception to 1 November 2021. In addition, the references in retrieved articles were searched manually to identify potential studies. 

### 2.2. Study Selection and Eligibility

EndNote X9 program (Version X9; Philadelphia, PA, USA) was used to import articles and remove duplicates. Two reviewers (Qianqian Chen and Wenjie Li) independently screened the titles and abstracts of these studies for relevance. Shortlisted full texts were screened against the eligibility criteria and appraised for quality before they were included in the review.

A study was included if:

(1) It was related to COVID-19;
(2) The study sample consisted of women with healthy babies in the postpartum period;
(3) The prevalence of PPD, and the factors associated with PPD were reported.

Studies were excluded if: 

Only the clinical outcomes of pregnant women were reported;

(1) Insufficient information was provided to calculate the prevalence and standard error of postpartum depressive symptoms;
(2) The screening tools for depressive symptoms was not validated;
(3) PPD was measured other than the Edinburgh postnatal depression scale (EPDS) with a cut-off score of 13 (defined as major depression symptoms) in order to maximize data uniformity across studies;
(4) Letters, case reports, or reviews were excluded.2.3. Data collection procedure.

### 2.3. Data Extraction and Quality Assessment 

Reviewers independently extracted the following data from the eligible studies: author; year of publication; country of study; study design; participant characteristics; sample size; the postpartum depression assessment criteria, cut-off points. We also extracted the PPD prevalence and the risk factors associated with it (when available). The Joanna Briggs Institute’s (JBI) critical appraisal instruments for the cross-sectional study were used to evaluate the quality of the included articles. This tool assessed the trustworthiness, relevance, and results of the published paper [23]. The JBI critical appraisal instruments for the cross-sectional study consisted of eight items. Each item is scored as 3, 2, or 1, and the total score was translated into a percentage. A quality score above 80% indicating high quality was included in the meta-analysis. The quality scores of each study were rated by two reviewers (Qianqian Chen and Wenjie Li) independently, with disagreements resolved by a third author (Juan Xiong).

### 2.4. Statistical Analyses

All analyses were performed using Review Manager 5.4 (The Cochrane Collaboration, London, UK). Statistical significance was defined as a two-sided *p*-value of less than 0.05. The pooled prevalence rate of postpartum depression and its 95% confidence interval (CI) were calculated by the random effect model of Dersimmonian and Laird. The Cochran’s Q-test and $I^{2}$ statistics were used to assess the heterogeneity across studies. Sensitivity analysis was conducted to evaluate the effect of the included study on the prevalence rate of PPD and the robustness of the results from the meta-analysis.

## 3. Results

### 3.1. Search Results

**Figure 1:**
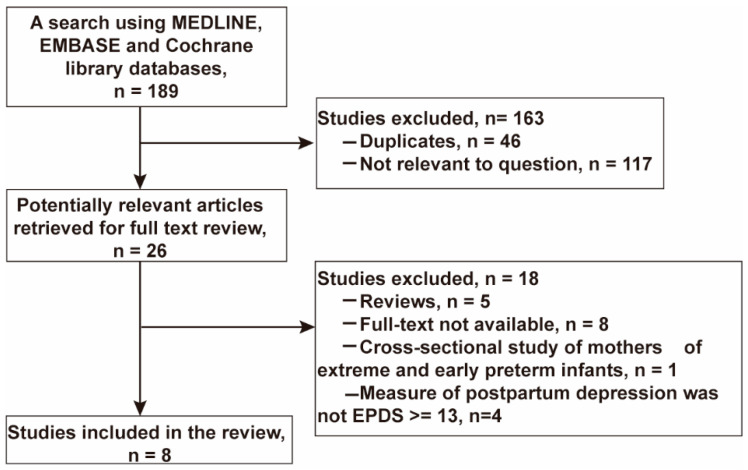
Study selection flow diagram.

A search using MEDLINE, EMBASE, and Cochrane library databases resulted in a total of 189 articles. No study was identified from the Cochrane library database. After removal of duplicates, 143 article titles and abstracts were screened for relevance. Of these, 26 articles were included for review in full-text for inclusion in the study. In this screening step, 18 studies were excluded for the following reasons: review articles (n = 5), full-text not available (n = 8), cross-sectional study of mothers of extreme and early preterm infants (n = 1), measure of postpartum depression was not EPDS (n = 4). A total of eight articles were included in this meta-analysis. The article selection flow diagram is presented in Figure 1.

### 3.2. Characteristics of the Included Studies

A total of eight studies [24,25,26,27,28,29,30,31] performed with 6480 postpartum women during the COVID-19 pandemic were included in the current systematic review and meta-analysis. All included studies utilized a cross-sectional study design and examined women up to 18 months postpartum. Most studies were conducted in developed countries, such as in the UK, Europe, and Canada. All eight studies used the EPDS with a cut-off score of 13 to define postpartum depression. More details of the included articles are listed in Table 1.

### 3.3. Prevalence of PPD during the COVID-19 Pandemic

**Figure 2:**
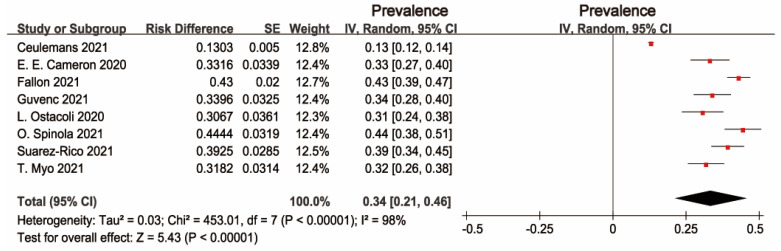
Forest plot of prevalence estimates of postpartum depressive symptoms.

**Figure 3:**
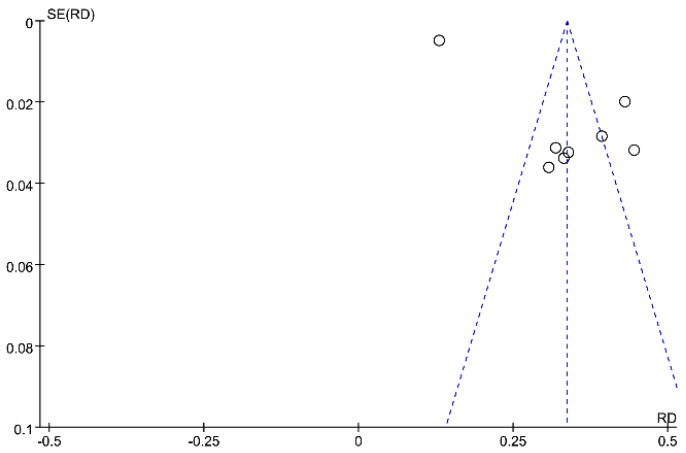
Funnel plot of prevalence estimates of postpartum depressive symptoms.

The pooled prevalence of PPD was 34% (95% CI: 21–46%), and high heterogeneity was detected across studies ($I^{2}$ = 98%, *p* < 0.001). Figure 2 presents the individual effective size of the studies that reported the prevalence of PPD among postpartum women during the COVID-19 period. The publication bias was evaluated by visual inspection of the asymmetry of the funnel plot. The funnel plot in Figure 3 exhibits an asymmetry pattern, indicating potential publication bias. 

**Figure 4:**
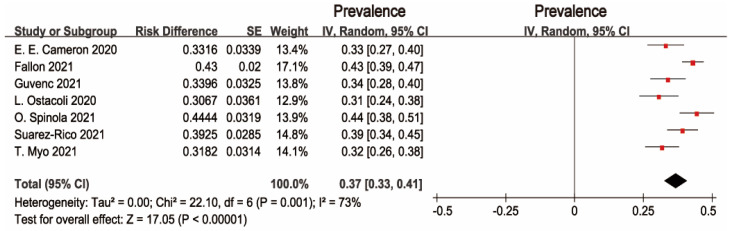
Forest plot of prevalence estimates of seven studies of postpartum depressive symptoms.

**Figure 5:**
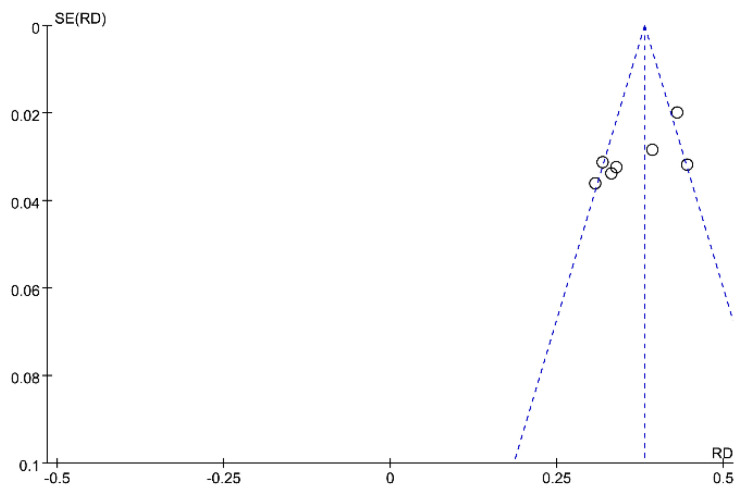
Funnel plot of seven studies of postpartum depressive symptoms.

Interestingly, a multinational cross-sectional study by Ceulemans reported that the prevalence of PPD was 13.03%, which was much lower than the others studies. Subsequent sensitivity analysis was conducted to evaluate the effect of this study. After excluding Ceulemans’s study, there was moderate heterogeneity across the seven studies ($I^{2}$ = 73%, *p* = 0.001). The pooled prevalence of depression was 37% (95% CI: 33–41%), as shown in Figure 4. The symmetry of the funnel plot indicates no publication bias (see Figure 5).

### 3.4. Risk Factors for PPD during the COVID-19 Pandemic

Risk factors for PPD were measured in the included studies, such as socio-demographic and clinical characteristics, stress and anxiety, lack of various supports, and the COVID-19 related factors. In terms of socio-demographic and clinical factors, maternal age [24], employment status [27,29], marital status [31], country [28], preterm delivery [25], pregnancy intention [25], breastfeeding status [27], and smoking [28] were reported to be associated with PPD during the COVID-19 pandemic. There were five studies that found that stress and/or anxiety caused by stressful events, perceived pain, low parenting competence, avoidant attachment style, and the physical and mental diseases negatively affected PPD [24,26,29,30,31]. Furthermore, various kinds of supports, such as frequency of maternal care received from health professionals, economic support received from family members, emotional support, and travel time to reach health center; were identified to positively influence PPD [25,27,29,30]. Moreover, several COVID-19 related variables, i.e., fears of infection were found to have a significant effect on the EPDS scores [24,26,29].

## 4. Discussion

To the best of our knowledge, it is the first systematic review and meta-analysis to assess the influence of the COVID-19 pandemic on the prevalence of PPD and its risk factors for postpartum women. Altogether, eight articles of 6480 postpartum women were included during the postpartum period. Given the availability of research for the present review, most of the studies were from developed countries, indicating that the review findings were poorly representative of women in developing countries. Therefore, much more research on maternal psychological wellbeing was strongly recommended to be undertaken in the middle- and low-income countries during the COVID-19 pandemic. Investigating the prevalence of depression symptoms among women in the postpartum period could shed some light on their mental and emotional states after delivery during the COVID-19 pandemic so that more attention and support from health professionals and policymakers could be offered to improve the maternal and infant outcomes [21]. 

The results from the present meta-analysis found that the pooled prevalence of major PPD (EPDS score 13 or higher) was 34% (95% CI: 21–46%) among postpartum women during the COVID-19 pandemic, much higher than the incidents of previous research during the non-pandemic period. For instance, the worldwide incidence of PPD until 2017 had been reported in the previous review were approximately 10% in the developed countries; and about 21–26% in the developing countries during the pre-COVID-19 period [8,9]. Another systematic review showed that the prevalence of PPD was 14.4% in the mainland of China before the pandemic [32]. The previous cross-sectional survey found the incidence of major PPD among Canadian women was 8.7% before the pandemic [33]. The other prior study reported the prevalence of PPD among 82,489 Japanese women was 14.0% during the pre-pandemic time [34]. 

The research findings indicated that the COVID-19 pandemic could make detrimental effects on maternal mental wellbeing among women after childbirth. Some factors could be attributed to it. First of all, social isolation associated with pandemics was identified to negatively influence the mental health and psychological wellbeing of postpartum women [17,18,19,35,36]. Moreover, routine health work interruption caused by the COVID-19 pandemic could negatively influence maternal physical and mental wellbeing [37]. For example, during the COIVD-19 pandemic, the face-to-face postpartum home visits were suspended in various countries, and alternative online consultation and guidance had to be provided from health workers via WeChat, phone, or video [38]. The limited access to routine maternity care during the pandemic was likely to further exacerbate the poor mental health of women [39]. 

It was noted that the prevalence of PPD (34%) in the study was higher than the incidents ranging from 10% to 22% in the other meta-analytic reviews [20,21,22] during the COVID-19 pandemic. One reason for inconsistent research findings could be that the previous evidence was all from the first wave of the infectious disease outbreak. To be specific, all articles in the previous review were published no later than 2020. As the outbreak continued, the impact of the ongoing/severe COVID-19 pandemic on maternal mental health was increasingly growing. Moreover, the previous meta-analytic reviews [20,21,22] used highly diversified tools and cut-off scores for PPD, which could cause results’ bias. Prior research likewise found that during disasters or events, the prevalence rates of mental disorders among postpartum women are significantly higher than those among the general population [20]. Thereby, various support measures and effective tailored interventions should be strongly recommended to be designed and conducted for postpartum women to improve their mental health, especially during the COVID-19 pandemic. Moreover, studies before the COVID-19 show that about 30% of PPD women could take more than 1 year to recover from depression, and there is no evidence whether the COVID-19 may affect the recovery time, further studies need to look at this aspect to check the persistence of depression.

As PPD could cause adverse maternal and infant outcomes [7], identified risk factors for PPD across different studies could help to design the targeted intervention strategies to prevent long-term impacts of the COVID-19 pandemic on maternal well-being and child development [31]. In the review, risk factors for PPD during the COVID-19 pandemic were defined as socio-demographic and clinical characteristics, stress and anxiety, lack of various supports, and the COVID-19 related factors. In terms of the influence of socio-demographic and clinical characteristics on PPD, the inconsistent results have been existant in the literature, which need to be explored in further research. For example, maternal employment status was likewise identified to affect maternal mental health in the recent review [26] and some previous studies [27,30]. By contrast, there were various studies reporting that employment status did not influence PPD [40,41]. 

In the current meta-analysis, five studies found that the status of stress and/or anxiety caused by the physical and mental diseases, the low parenting competence, and the avoidant attachment style negatively affected PPD during the pandemic [24,26,29,30,31], which was consistent with the previous research [26]. For instance, women with poor physical or psychological conditions have previously been identified as a risk factor for PPD [42]. The limited access to health services during the pandemic may have prevented women with physical or mental illnesses from seeing clinicians, potentially contributing to exacerbated psychological stress and anxiety [37,43]. Moreover, women with a lower parenting competence [1] and an insecure attachment style [30] were prone to suffering from much more parenting problems leading to a higher level of stress and anxiety in women, which negatively impacted the wellbeing of infants and mothers. 

In terms of the positive effect of various supports on PPD, it already has been well documented in the literature [25,29,30]. According to the social exchange theory [44], different kinds of supports such as informational support, material support, emotional support, and evaluation of the support provided by health professionals and family members could promote women’s ability to manage their depression symptoms. However, during the pandemic, various supports were seriously limited owing to the restrictions that have been put into place to decrease the risk of transmission of COVID-19 [45]. Thus, special support measures from health professionals and policymakers should be designed and conducted to improve maternal and infant outcomes during the lockdown period. 

The COVID-19 related variables, i.e., fears of infection for themselves, their babies, and their families, were found to have a significant effect on EPDS scores [24,26,29]. Due to fear of infection transmission, postpartum women were reported to ask for discharge from the hospital as soon as possible after childbirth, avoid going to the hospital for routine postpartum examinations, and state that they did not accept home visits by health professionals in the postpartum period [26]. The fears experienced during the COVID-19 pandemic had negative psychological consequences that increased both the risk of PPD and the level of anxiety [17,45]. Just like the WHO Director-General said: “fear from the virus is spreading even faster than the virus itself” (p. 129) [46]. These findings indicated that the COVID-19 pandemic, as an acute public health issue, indeed required the continued, comprehensive, and long-term health education to effectively alleviate women’s panic and fear, thus improving the mental wellbeing of this vulnerable population and the associated outcomes.

The major strength of the review is to maximize data uniformity across studies as the included research applied the same type of screening tool and the cut-off score commonly used in the postpartum setting of different countries. Some limitations of the current review need to be stated. Firstly, our review only focused on studies published in the English language, which may have resulted in the exclusion of potentially relevant studies from non-English speaking countries. Secondly, our systematic search using mainstream research databases could cause the lack of the relevant grey literature or studies published in local specific journals. Thirdly, even though an extensive search using all possible search terms was conducted by the researchers, it was not impossible that some eligible studies could have been omitted. Fourthly, all studies were cross-sectional, so the effects of the COVID-19 pandemic in the longer terms need additional longitudinal studies. Fifthly, most studies in the review were from developed countries, indicating that the findings were poorly representative of women in the middle- and low-income countries. Thus, further research on maternal psychological wellbeing during the COVID-19 was strongly recommended to undertake in developing countries. 

## 5. Conclusions

This review is unique in its contribution and comparisons of the pooled prevalence of PPD and its risk factors in the context of the COVID-19 pandemic, which may help guide the tailored and preventive intervention. The pooled prevalence of PPD in the review was 34% (95% CI: 21–46%) during the COVID-19 pandemic, much higher than the incident of previous research during the non-pandemic period. Risk factors for PPD during the COVID-19 pandemic were defined as socio-demographic and clinical characteristics, stress and anxiety, lack of various supports, and the COVID-19 related factors. The research findings indicated that the COVID-19 pandemic could make detrimental effects on maternal mental wellbeing among women after childbirth. Investigating the prevalence and risk factors of PPD among postpartum women could shed some light on their mental and emotional states after childbirth; so that support measures and tailored interventions from health professionals and policymakers could be offered to improve the maternal and infant outcomes, especially during the COVID-19 pandemic. Much more research on maternal psychological wellbeing was strongly recommended to undertake in the middle- and low-income countries during the COVID-19 pandemic. As an acute public health issue, the COVID-19 pandemic indeed required the continued, comprehensive, and long-term health education to effectively alleviate women’s panic and fear, thus improving the mental wellbeing of this vulnerable population and the associated outcomes.

## Figures and Tables

**Table 1:** Characteristics of included studies on PPD and its risk factors during the COVID-19 pandemic.

Study	Year	Country	Postpartum Time	n	N	Prevalence (%)	95% CI	Study Period	JBI Score (%)	Risk Factors
Suarez-Rico. et al. [24]	2021	Mexico	1–3 months	115	293	39.25	(33.62, 45.10)	August 2020–September 2020	91.67	Maternal age; COVID-19: positive SARS-CoV-2 test; Perceived stress; Anxiety
Myo et al. [25]	2021	Myanmar	≤6 months	70	220	31.82	(25.72, 38.42)	April 2020–May 2020	100	Preterm delivery; Pregnancy intention; Frequency of antenatal care received; Travel time to reach health center; Birth interval more than 5 years
Guvenc et al. [26]	2021	Turkey	1–1.5 months	72	212	33.96	(27.62, 40.76)	May 2020–July 2020	91.67	COVID-19: Fear of being infected; Fear of transmission to the baby; Anxiety
Fallon et al. [27]	2021	UK	0–3 months	264	614	43.00	(39.04, 47.02)	April 2020–May 2020	95.83	Less emotional support: perceived psychological change as a result of social distancing measures; Breastfeed status; Parenting competence; Employment status
Ceulemans et al. [28]	2021	Ireland, Norway, Switzerland, the Netherlands, and UK	0–3 months	592	4542	13.03	(12.07, 14.05)	June 2020–July 2020	95.83	Country; Smoking; Chronic somatic and mental illness
Spinola. et al. [29]	2020	Italy	0–12 months	108	243	44.44	(38.09, 50.93)	May 2020–June 2020	95.83	COVID-19: fear of infection (for others and child); Socio-economic status; Received economic support from family; Previous emotional troubles
Ostacoli et al. [30]	2020	Italy	0–3 months	50	163	30.67	(36.41, 52.15)	June 2020–June 2020	100	Perceived pain; Support by healthcare staff during childbirth; Avoidant attachment style
Cameron et al. [31]	2020	Canada	0–18 months	64	193	33.16	(26.57, 40.28)	April 2020–April 2020	95.83	Stressful events past month; marital status

## Data Availability

The data presented in this study are available on request from the corresponding author. The data are not publicly available due to privacy restrictions.
